# Psychological Aspects to Consider in Breast Cancer Diagnosis and Treatment

**DOI:** 10.1007/s11912-021-01049-3

**Published:** 2021-03-11

**Authors:** Loredana Dinapoli, Giuseppe Colloca, Beatrice Di Capua, Vincenzo Valentini

**Affiliations:** 1 Dipartimento di diagnostica per Immagini, Radioterapia Oncologica ed Ematologia, Fondazione Policlinico Universitario A. Gemelli IRCCS, Largo A. Gemelli 8, 00168 Rome, Italy; 2 UOS Psicologia Clinica, Fondazione Policlinico Universitario A. Gemelli IRCCS, Roma, Italia; 3 Catholic University of the Sacred Heart, Rome, Italy; 4 Università Cattolica del Sacro Cuore Istituto di Radiologia, Roma, Italy

**Keywords:** Breast cancer, Elderly, Personalized medicine, Distress, Depression, Quality of life

## Abstract

**Purpose of Review:**

Breast cancer (BC) is the most common cancer diagnosed in women in the West World. Coping with cancer is cause of extreme stress for patients and their family. The purpose of this review is to evaluate possible approaches to follow to control those situations that can impact on quality of life (QoL) and compliance to treatments.

**Recent Findings:**

Anxiety, distress, depression, and posttraumatic stress disorder are the most frequent psychological disorders in BC patients*.* Cognitive disorders and sexual dysfunction can also be important in affecting QoL both in younger and older patients. Younger and older patients show different characteristics of these disorders and different strategies of managing them.

**Summary:**

Several psychotherapeutic and supportive approaches have proven effective in managing psychological disorders in BC patients. Every BC patient should be supported with these techniques during her entire oncological history, in order to increase QoL and compliance to treatments.

## Introduction

Breast cancer is the most common noncutaneous malignancy diagnosed in women in the West World with one in eight women developing breast cancer (BC) in her lifetime [1]. Localized disease accounts for 61% of all BC diagnosed in the USA, and the 5-year survival rate for this population approaches 98% [2]. In Italy, BC represents 22% of neoplasms diagnosed in elderly women [3].

Facing BC represents huge stress for the patient that has to deal with new and challenging issues and choices. Accepting diagnosis, undergoing treatments, understanding prognosis, handling possible side effects, managing a possible relapse, facing an uncertain future, are all stages of a stressful process that can cause psychological instability and can lead to depression or other mood disorders.

A large proportion of BC patients experience multiple concurrent psychological symptoms during their cancer care trajectory, such as distress, anxiety, depression, cognitive impairment, and body image/sexual dysfunction [4].

## Distress, Anxiety, and Depression

Anxiety is one of the most common psychological symptoms in BC patients, with rates ranging from 10 to 30% [5]. The patient can experience anxiety symptoms because of the anticipation of negative outcomes [6], and the uncertainty about the future; anxiety can spring from the concern over recurrence and the worry of treatment side effects both during and after treatments [7, 8]. Recent findings suggest that anxiety is even more prevalent than depression [9], in contrast to what has been presented in the past [10].

Distress is a broad construct, covering a wide continuum of emotions related to symptoms of depression, anxiety, and adjustment disorder [11]. Even though prognostic prospects are relatively good, a BC diagnosis is threatening and confronts women with numerous additional stressors, such as treatment and its side effects [12]. BC patients’ distress levels may rise at different stages throughout the disease trajectory [13]. For example, some patients experience clinical distress at the time of diagnosis, yet during the active phases of treatment, BC patients report higher distress when compared to prostate cancer patients, expressing concerns about fatigue, family and friends, weight, fears, worries, and pain [14]. Other BC patients are mostly affected after treatment due to the discontinuation of regular contacts with medical specialists and/or to treatment-induced side effects [13]. Among the sociodemographic factors, younger age, living with a partner and children or only with children, and having a paid job, predict a greater endurance of clinical distress. From an oncological point of view, having a mastectomy, chemotherapy or radiation, and chemotherapy compared to radiation therapy alone, were strong clinical predictors of durable distress [13]. In addition, having received psychosocial services before the BC diagnosis, and having two or more comorbid conditions are also strong predictors of long-lasting distress.

Among patients with all types of cancer, the prevalence of depression among those with BC is the third highest after pancreatic and head and neck cancer [5, 15]. The rate of depression in BC patients was estimated between 10 and 30%, depending on the study population, study design, and choice of depression measure [5]. It negatively affects women’s treatment regime, quality of life (QoL), self-care, and decreases immunity and chances of survival [16]. Diagnosis of depression can be challenging in cancer patients because symptoms of depression overlap with physical symptoms as a consequence of illness or treatment.

Both anxiety and depression have a tremendous impact on BC patients. The literature contains a disproportionate number of studies involving younger versus older patients, although the rates of depression after BC are similar between the two groups [17].

### Elderly BC Patients and Psychological Profile

In elderly BC patients, several factors may add psychological burden, such as a decreased reserve and social supports, clinical bias toward undertreatment and a particular sensitivity to effective medical communication [17]. Elderly women may be less disposed to see a psychiatrist or psychologist, or to seek medical help for any breast symptom. Instead, they report more concerns about disfigurement and financial consequences related to health care, resulting in a delay in cancer treatment of up to three months [18]. Regarding other psychological characteristics that could influence mood and distress in BC patients, the only paper exploring resilience in BC survivors did not report an influence of age on this construct [19]. On the other hand, a recent paper exploring coping styles among young and old cancer patients [20•] showed that older individuals have a resignation strategy. This results in higher levels of anxiety and depression, scarce cognitive strategies, a conviction of low control on events, low compliance, and an attitude of renunciation. This study does not support the previous data demonstrating older BC survivors had lower distress that younger BC patients [21]. Compared to younger BC survivors, older survivors are often diagnosed at a life stage frequently without multiple responsibilities as primary caregivers to their spouses or partners, children, and parents, and are often retired from work. Intuitively, older BC patients are less threatened by death because they cope better with major medical crises [21]. Moreover, comparing to younger patients, older patients have less fear of recurrence. Timing of onset and duration of symptoms are different too: younger survivors demonstrate an increase of fear during the first 1.5 years after surgery, while in older survivors, levels of fear are stable for the first 6 months after which it declined [22]. Although the data on distress, anxiety, and depression are not homogeneous, several factors, including age, education, social support, and treatment characteristics, appear to have an effect on patients after BC diagnosis. Particular attention should be paid to anxiety/depression in elderly BC patients because of the demonstration of specific periods of time and associated risk factors which can influence mood in older patients (Tables 1 and 2) [17].

**Table 1:** Periods of increased vulnerability for elderly patients with cancer

Finding a suspicious physical symptom	
During work-up	
Transition in treatments	
Treatment decision: should I treat the cancer or not (at my age?)	
Awaiting treatment	
Conclusion of treatment	
Stopping ineffective treatments and other medical decision	
Advanced cancer	
Discharge from hospital	
Recurrence	
Stresses of survivorship	
End of life

**Table 2:** Risk factors for developing anxiety and depression in elderly patients with cancer

Pain	
Fatigue	
Poor physical condition	
Comorbid medical conditions	
Need for assistance with activities of daily living	
Functional disability	
Poor eyesight	
Sensory losses	
Cognitive decline	
Lack of perceived control	
Other life stressors or losses	
Loneliness	
Fatalistic feelings	
Inadequacy of emotional support	

One of the challenges in facing these issues in elderly patients is the phenomenon that the number of studies on the effectiveness of psychooncological interventions for patients with breast and other cancers decrease with increasing age and are extremely limited among patients older than 60 years [23]. Possible interventions for elderly patients to reduce psychosocial problems are supportive psychotherapy, mindfulness-based stress reduction protocol, and cognitive behavioral therapy. The specific intervention described for geriatric cancer patients are coping and communication support for older cancer patients (CCS) and CARE intervention—cancer and aging: reflections of elders [24]. Also, web-based intervention (e-health) resulted in significant support for elderly BC patients during their upcoming chemotherapy and offered them effective coping strategies [25].

### Cancer and Posttraumatic Stress Disorder

For some patients their cancer experience is decisively traumatic, with psychological consequences that might result in posttraumatic stress disorder (PTSD) [26•]. PTSD is a mental disorder occurring after exposure to life-threatening episodes (criterion A) and is characterized by intense reliving of the traumatic event through intrusive memories and nightmares (criterion B); avoidance of reminders of the event (criterion C); negative alterations in cognitions and mood (criterion D); hypervigilance toward potential threats in the environment (criterion E); and in some cases, persistent or recurrent depersonalization symptoms [27]. Cancer patients often describe constant concerns, nightmares about the neoplastic disease or the treatment, and worries of recurrence and about the future. Intrusive symptoms are the most prevalent, with a rate of 11–45% [26•]. Evidence examining PTSD symptomatology among long-term cancer survivors shows rates of lifetime cancer-related PTSD that range from 3 to 22%, [26•].

Eye movement desensitization and reprocessing (EMDR) therapy is an eight-phase psychotherapeutic comprehensive approach that has been extensively researched and proven effective for the treatment of trauma [28]. According to the World Health Organization (2013), trauma-focused cognitive behavioral therapy and EMDR therapy are the only psychotherapies recommended for children, adolescents, and adults with PTSD. Two recent papers describe the efficacy of EMDR in cancer patients: Jarero et al. [29] performed a randomized clinical trial (RCT) investigating the eye movement desensitization and reprocessing Integrative Group Treatment Protocol adapted for ongoing traumatic stress (EMDR-IGTP-OTS) in women with cancer. Data analysis showed that the EMDR-IGTP-OTS was effective in significantly reducing symptoms of PTSD, anxiety, and depression, which was maintained at the 90-day follow-up. The standard EMDR Integrative Group Treatment Protocol for early intervention provides individual EMDR therapy in a group setting. The protocol provides EMDR’s eight phases [28] in a group therapy model with an art therapy format (e.g., drawings, symbols). It uses the Butterfly Hug (BH) method as a self-administered bilateral stimulation to process traumatic material. During the session, the patient draws a picture or symbol representing the traumatic events and then focuses on it for an average of 3 min while performing the BH method. This process is repeated four times in the session, with the patient usually reporting substantial decreases in the subjective disturbance at the session end.

Carletto et al. [26•] performed a quasiexperimental study comparing the neurobiological correlates of two different therapeutic interventions (EMDR versus treatment as usual—TAU) for treating cancer-related PTSD in women with BC. Participants received 10 EMDR sessions over a period that varied between 2 and 3 months. EMDR was administered in accordance with Shapiro’s protocol for traumatic events [28], and it specifically followed the EMDR specific protocol for oncological patients [30]. The TAU group received four sessions of supportive therapy, one every other week for 2 months. This consisted of standard treatment to support patients in coping with the psychological symptoms related to BC. Participants were assessed using EEG at two separate time points: at baseline (T0) and after the last session of EMDR or TAU treatment (T1). EMDR was found to be superior to TAU in reducing the proportion of patients who were classified as having a PTSD clinical diagnosis. EMDR resulted in a clinically significant improvement, achieving scores below the clinical threshold in intrusive, avoidant, and hyperarousal symptoms. Furthermore, EMDR was found to be effective in reducing depressive symptoms. Neither EMDR nor TAU was effective in reducing anxiety symptoms.

## Cognitive Impairment

Many BC survivors report changes in memory and overall cognition during or after chemotherapy or hormone therapies [31], often referred to as ‘Chemo brain’. Chemo brain (CB) (or chemotherapy-induced cognitive impairment CICI) includes impairment of a patient’s memory, learning, concentration, reasoning, executive function, attention, and visuospatial skills during and after the discontinuation of chemotherapy [31]. These symptoms have significant negative implications on activities of daily life, including driving, paying bills, and reading dense or ‘technical’ material [32]. Although CB is frequently associated with treatment, the cause of this perceived decrease in cognitive function is unclear: some potential factors are systemic chemotherapy or adjuvant hormone therapies (aromatase inhibitors or tamoxifen), or the association with a cancer diagnosis. For example, BC patients often present acute stress disorder, depression, and anxiety disorders all of which can worsen cognitive function [31]. Numerous studies investigated the relationship between hormone/aromatase inhibitors or chemotherapy and perceived reduction in cognitive ability. These studies showed that, often, these symptoms are not supported by formal cognitive testing [31], and this has led many researchers to approach CB by addressing real cognitive loss versus perceived cognitive loss [33, 34]. These studies have relied on small sample sizes, included heterogeneous disease and treatment groups, lacked pretreatment chemotherapy assessments, and used normative control data [35••]. Even if only perceived, CB significantly alters the QoL and should be considered as such when assessing BC patients’ needs. One suggestion for the general oncology practice is that older patients should be evaluated for frailty and cognitive reserve [36, 37]. Several brief screening tools for orientation, mental status, and basic cognitive functions are available and can be easily administrated, but they lack sensitivity to detect subtle cognitive changes [38]. For this reason, extensive neuropsychological evaluation of verbal memory (short and long term) and nonverbal memory executive functions, mental processing, language, and attention is recommended using written tests to assist in distinguishing cancer treatment–related problems from incipient dementia [38].

As the field of geriatric oncology moves forward, it will be important to ensure that series of tests are valid and have an adequate standardization. The results of these assessments could then be useful as adjunct tools for identifying subgroups of older patients who are likely to be at the highest risk for cognitive decline after systemic therapy.

Treatment options for the older patient includes cognitive rehabilitation training, which strengthens and restores cognitive abilities by means of cognitive drills. Regarding BC patients, several studies demonstrated good results but only at a younger age [39, 40]. Compensatory remediation techniques are simpler than cognitive rehabilitation and offer strategies for everyday life, e.g., the use of calendars, planners, and timers.

The development and delivery of home-based or web-based interventions may have advantages over traditional clinic–based interventions. However, there is a scarcity of innovative interventions for cognitive training [41•]. A recent review showed that, to date, none of these were specifically developed for BC patients [41•].

Regarding psychopharmacological interventions, studies on methylphenidate and modafinil showed mixed results [38], therefore larger studies are warranted to assess the validity of drug effects on cognitive performances.

## Body Image and Sexual Dysfunctions

Body image (BI) is conceptualized as a multifaceted construct, defined as “the mental representation of one’s body, thoughts, and feelings about one’s physical appearance, attractiveness, and competence, as well as one’s perceived state of overall health wholeness, functioning, and sexuality” [42]. A diagnosis of BC is likely to further exacerbate the women’s propensity to focus on body image–related evaluation and investment [43]. Losing a breast is inherently linked to a woman’s identity, sexuality and sense of self, with approximately one-third of BC survivors expressing distress directly related to disturbed body image after successful cancer treatment, particularly among younger women [44]. Although BI disturbances usually improve over time, the subgroup of BC survivors continues to experience BI-related concerns despite breast conservation or reconstruction techniques [42].

Body image has been validated to be associated with women’s sexuality [45]. Amongst BC patients, 73.4% have sexual dysfunctions, suggesting that women with BC constitute a high-risk group [46]. This may be due to BC-specific treatment experiences, such as BI changes after breast surgery, hormone treatments, changes in hormone levels after ovariectomy, and the physiological and psychological effects of chemoradiotherapy. Attention should be paid to changes in sexual function affecting women with BC [46]. Natural menopause, drugs, or surgical castration can all contribute to reduced estrogen levels in women with BC, and these reduced levels can induce or aggravate sexual dysfunction ultimately leading to a reduction in sexual desire, reduced sexual arousal, lack of vaginal lubrication, pain during intercourse, difficulty achieving orgasm, and genital hypoesthesia [46]. Women’s intrapsychic experience of changes to sexuality includes a fear of loss of fertility, negative body image, feelings of sexual unattractiveness, loss of femininity, as well as alterations to a sense of sexual self; the impact of such changes can last for many years after successful treatment and can be associated with serious physical and emotional side effects [47]. Even if sexuality is mostly investigated in young patients, it is important to show that approximately 53% of adults between the ages of 65 to 74 are sexually active [48]. Sexuality is one of the first elements of everyday life influenced by cancer and unlike other side effects that tend to improve over time, survivors’ untreated sexual problems typically persist or worsen [49]. The causes of sexual dysfunction in elderly patients are related to physical changes (alopecia, hormone imbalances, pain), psychological factors (adopting the “patient role”—asexual—, altered body image, fear of death, rejection by partner), and social factors (communication difficulties regarding sexuality) [48]. Some factors can be exacerbated in the older patient, such as difficulty of speaking about sex, fear of death, or reassignment of priorities. The treatment of sexual dysfunction in the older patient includes estrogen supplements, vaginal lubricants, change in antidepressants (SSRIs can delay orgasm), dilators in conjunction with Kegel exercises, a referral to a sexual therapist, and couple-based interventions [48]. Esplen [42] conducted an RCT to assess the efficacy of an 8-week group for women after BC treatment. The manual-based intervention combined two steps: expressive guided-imagery exercises integrated within a model of group-therapy principles. This intervention facilitates exploration of identity, the development of new self-schemas, and personal growth. The intervention also included an educational component on the social and cultural factors affecting women’s self-esteem and BI. Patients in the intervention group showed significantly less concern/distress about body appearance, decreased body stigma, and a lower level of BC-related concerns, compared with women in the control group. BC-related QoL was also better in the intervention group compared with the control group at the 1-year follow-up. There was no statistically significant difference in sexual functioning, indicating that more studies are needed to improve sexual health in BC patients. A review on BI in cancer [50] presented and patient-doctor communication found that BI difficulties were found across patients with diverse cancers, and were most prevalent in the immediate postoperative and treatment period. Age, body mass index, and specific cancer treatments have been identified as potential risk factors for body image disturbance in cancer patients. In addition, specific interventions were described (Table 3). The authors also proposed practical strategies for the oncologic health care team when addressing BI concerns, which is referred to as The Three C’s (common, concerns, consequences) [51]. This strategy encourages patients to discuss their BI concerns, thereby allowing the health care team to identify emotional difficulties and to develop a solution. At the beginning of a clinical encounter, providers should remind patients that BI difficulties are very common to normalize their concerns and reduce shame, embarrassment, and stigma. Then, providers, should ask about specific concerns which may include impending treatment effects or recent or prolonged changes to the appearance and/or functioning. This step is accomplished with open-ended questions that elicit patient narrative. Finally, therapists ask patients about consequences of their BI difficulties on daily functioning, with particular attention to social, emotional, and occupational problems. Regarding BI, a recent study [52•] investigated the effects of a brief beauty care intervention on self-reported symptoms of depression, QoL, BI, and self-esteem in 39 female BC patients with appearance-related treatment side effects. The intervention consisted of a single-session group makeup workshop with an incorporated portrait and upper-body photo shooting. Photos were also professionally edited and sent to the patients. While groups did not differ regarding any measure at the pretreatment baseline assessment, patients in the intervention group reported fewer symptoms of depression, higher QoL, and higher self-esteem compared with baseline. Follow-up at 8 weeks indicated moderate stability of these improvements, in contrast to previous research, indicating beneficial short-term and mid-term effects of beauty care on psychological outcomes in patients with early BC.

**Table 3:** Body image intervention studies

Cognitive-behavioral therapy interventions	
• Therapeutic approach that targets dysfunctional cognitions, emotions, and behavior by alteration of cognitions • Components included psychoeducation, stress management, problem-solving, cognitive reframing, and communication skills training	
Other psychological interventions	
• Psychosexual therapy focusing on communication training, sensate focus, and body image exposure • Expressive-supportive therapy focusing on expression of thoughts and emotions, receiving and offering support, coping skills	
Education interventions	
Information disseminated in lecture formats to increase knowledge on disease and treatment with the aim of increasing self-efficacy	
Cosmesis-focused interventions	
• Education on using cosmetics to improve appearance • Provision of beauty treatment regimens (manicure and pedicure, hairdressing, make-up)	
Sensate-focused/physical fitness interventions	
• Massage therapy with the aim of stress reduction • Hatha yoga focusing on changing patient’s perceptions about physical constraints imposed on their body • Strength training and physical exercise to regain physical fitness	

## Conclusion

Elderly and young BC patients have similar psychological problems related to trauma of diagnosis, side effects of therapies with the consequent change of body image and sexual behavior, fear of recurrence, and end of life. Many psychotherapeutic and supportive approaches have proven effective in various patient groups. In order to ensure a successful psychological approach in elderly patients, the most effective interventions include:

- Psychotherapy with a soft body mediation approach;
- Meditative and reconciliation interventions with one’s own spiritual well-being and needs;
- Soft cognitive rehabilitation interventions not too demanding for patients;
- Interventions for the elaboration of the group trauma with EMDR.
